# Functional Biomarkers Associated with Risk of Low Back Pain in Firefighters: A Systematic Review

**DOI:** 10.3390/jfmk10040441

**Published:** 2025-11-14

**Authors:** John M. Mayer, Mina Botros, Elizabeth Grace, Ram Haddas

**Affiliations:** 1 The Vert Mooney Research Foundation (dba U.S. Spine & Sport Foundation), San Diego, CA 92074, USA; 2 Department of Orthopaedics and Physical Performance, University of Rochester Medical Center, Rochester, NY 14642, USA; 3 Edward G. Miner Library, University of Rochester Medical Center, Rochester, NY 14642, USA

**Keywords:** low back pain, firefighters, occupational health, functional biomarkers, musculoskeletal injury, prevention, biomechanics, physical fitness

## Abstract

**Background:** Firefighters are at elevated risk of low back pain (LBP), yet predictors, mechanisms, and interventions for LBP in this occupation remain poorly defined. The purpose of this study was to systematically review the literature and synthesize the evidence on functional biomarkers associated with the risk of LBP in firefighters. **Methods:** PubMed, EMBASE, CINAHL, and PEDro were searched for studies evaluating functional biomarkers in firefighters with or without LBP, including aerobic capacity, anthropometric measures, disability/kinesiophobia, functional work tasks/capacity, imaging/structural/morphological characteristics, kinematics, movement quality/range of motion, muscular fitness, overall physical fitness, physical activity. Empirical evidence statements were generated for each biomarker domain, under Protocol Registration PROSPERO (CRD420251010061). **Results:** Eighteen studies (*n* = 32,977) met inclusion criteria and were predominantly cross-sectional (14/18) with fair quality (13/18), which suggests a substantial risk of bias. Higher disability/kinesiophobia and poorer functional work task performance were linked to increased risk of LBP, although causal relationships cannot be determined. Associations for the eight other biomarkers were inconsistent. Two interventional studies demonstrated benefits from trunk-focused exercise. **Conclusions:** The literature examining functional biomarkers and LBP in firefighters is fragmented, which precludes making robust and broad clinical recommendations for evidence-based implementation. Findings of future research may ultimately lead to approaches to improve the safety and health of firefighters with LBP through patient-centered and tailored programs addressing integrated functional biomarkers across the continuum of prevention, clinical care, and resilience development.

## 1. Introduction

Firefighting is a physically demanding and psychologically stressful occupation, which puts firefighters at risk for a wide variety of health disorders [1]. Musculoskeletal injuries are notably troublesome in this population and negatively affect job performance and preparedness, quality of life, activities of daily living, physical function, and behavioral health [1,2]. Furthermore, musculoskeletal injuries are common in firefighters, accounting for 43–62% of all work-related injuries and occurring more frequently than burn, thermal, and inhalation injuries [3].

Among the many categories of musculoskeletal injuries and related disorders, low back pain (LBP) is especially burdensome in these tactical athletes [4]. For example, the prevalence of LBP in firefighters is high, with 45–86% reporting a lifetime prevalence [1,5,6], and approximately 20% reporting that they are currently working with LBP [7]. Firefighters with LBP typically have longer periods of absence from work and incur higher medical costs than those resulting from other types of injuries [1,4]. Furthermore, LBP is a primary reason for disability and early retirement in firefighters [1,4,7].

Various biopsychosocial health domains (e.g., kinematics, physical fitness, anthropometric measures, work environment and exposures, behavioral health, sleep) have been shown to be associated with LBP and other musculoskeletal injuries in firefighters [7,8,9,10]. With these relationships in mind, interventions for musculoskeletal injuries have been applied to the fire service and some successes have been reported in observational studies [11,12] and a controlled trial [6]. However, implementation of worksite musculoskeletal health programs for this occupation has substantial barriers [13]. While it is plausible that focusing on high-risk firefighters and customizing programs according to risk levels could facilitate adherence, clinical outcomes, and cost-effectiveness [14], this area has not been fully explored.

The aforementioned research has provided useful information about risk factors for general musculoskeletal injuries in this population, yet the relationship between functional biomarkers (e.g., biomechanical, functional performance, physical fitness, anthropometric) and the risk of LBP in firefighters has not been systematically reviewed. Hence, gaps in knowledge exist that preclude generalizability and informed decision-making about implementing tailored interventions and countermeasures for this disorder.

Assessing LBP as a distinct entity from other musculoskeletal injuries is critical for several reasons. For example, LBP is consistently identified as a leading cause of disability worldwide [15], with substantial health and socioeconomic consequences in the fire service [1,4]. Despite this impact, LBP is often grouped together with other musculoskeletal injuries (e.g., shoulder, knee, and other regions) in firefighters in both clinical assessments and research designs, limiting the ability to isolate its unique biomechanical, psychosocial, and physiological contributors. From a clinical standpoint, the spine is biomechanically and neurologically distinct from other musculoskeletal structures [16]. Therefore, effective management of LBP requires approaches specifically designed for spinal health, rather than generic musculoskeletal assessments [17], and clinical practice guidelines have been produced by major health professional associations specifically for LBP [18,19].

Previous research suggests that LBP represents a physiological protective mechanism [20] and indicates a maladaptive pathological process when it persists [21,22]. Thus, LBP in firefighters could arise from an interplay of biomechanical, psychosocial, and environmental stressors, with adaptive responses that may either protect or predispose individuals to chronic dysfunction [22]. Furthermore, this dysfunction should be interpreted and addressed within the context of unpredictable, high-intensity, and unscheduled physical demands of firefighters [23,24]. However, these concepts have not been systematically examined in firefighters.

The purpose of this study was to systematically review the literature and synthesize the evidence on functional biomarkers associated with the risk of LBP in firefighters.

## 2. Materials and Methods

### 2.1. Study Design, Reporting, and Registration

A systematic review of the scientific literature was conducted using methods adapted from previous reviews on LBP [25,26,27,28]. The findings were reported according to the Preferred Reporting Items for Systematic Reviews and Meta-Analyses (PRISMA) guidelines [29], and the PRISMA checklist is depicted in Table A1 of Appendix A. The review was registered with the International Prospective Register of Systematic Reviews (PROSPERO) (CRD420251010061), which contains key details of the protocol, including background and significance, search strategies, review processes, methods, and analyses. A full protocol is not available elsewhere and no meaningful amendments were made to the protocol provided in the registration.

### 2.2. Eligibility Criteria

Condition being studied: LBP in firefighters.

Participants/population: Studies that assessed firefighters were included, as defined by the U.S. Bureau of Labor Statistics [30]: “Firefighters control and put out fires and respond to emergencies involving life, property, or the environment.” Firefighters of any employment status (e.g., career, volunteer), type (e.g., structural firefighting, wildland firefighting), and location were included.

Intervention/Exposure: LBP was assessed considering the various occupational hazards faced by the target population (firefighters), leading to this condition that may be influenced by functional biomarkers and related characteristics. Firefighters with a history of LBP served as cases. The operational definition of LBP is [6,31,32] “… an injury (e.g., sprain, strain, trauma) or illness (e.g., infection, neurologic disorder) that resulted in pain or discomfort below the rib cage and above the lower buttocks.” Studies that used any of the following terms to describe LBP were included: back pain, back injury, back injuries, LBP, low back injury, low back injuries, lower back pain, lower back injury, and lower back injuries. Studies that assessed LBP using patient-reported outcome measures or administrative data, such as workers’ compensation claims and costs, were included.

Comparator/Control: Firefighters without a history of LBP served as comparators/controls. Also, for eligible one-arm interventional studies with more assessments at two or more time points, firefighters served as their own controls. Studies that did not compare firefighters with a history of LBP to those without a history of LBP were excluded.

Outcomes: Dependent variables—The authors could not find a standard and published definition of functional biomarker in the context of musculoskeletal disorders, particularly for LBP in high-risk occupations. Thus, the term functional biomarker was broadly defined and consisted of heterogeneous variables that were arbitrarily categorized into domains. The conceptual framework behind the domains was based on the authors’ experience with research, clinical, and evidence-based practices, and the authors’ consensus process. For the purpose of this research, a pragmatic operational definition of functional biomarker is an “objective, quantifiable measure that reflects changes in the body’s function related to the pain condition or its response to treatment” that “serves as an indicator of a person’s ability to perform everyday tasks or that reflects the underlying biological and neurological processes related to function, physical fitness, and disability” (Google AI, response to question from author, 4 November 2025). To align with this review’s primary aim and to avoid being overly broad, the operational definition of functional biomarker does not include other dimensions within the biopsychosocial model, such as psychosocial and cognitive factors.

Various quantitative measures assessing functional biomarkers and related characteristics were included, such as motion analysis, electromyography, exercise tests, functional capacity tests, physical fitness tests, and anthropometric measures (e.g., body fat, waist circumference, body mass index). Patient-reported outcome measures for function and disability were also included. For analysis, outcomes were categorized in the following domains: aerobic capacity, anthropometric measures, disability/kinesiophobia, functional work tasks/capacity, imaging/structural/morphological characteristics, kinematics, movement quality/range of motion, muscular fitness, overall physical fitness, and physical activity.

Independent variable—Pain measures (i.e., patient reported outcome measures, administrative data) were used to characterize the case (with LBP) versus control (without LBP). Additionally, interventions aimed at addressing the functional biomarkers described above for prevention or treatment of LBP were considered.

Time: Any duration or frequency measurement of the exposure or intervention period was included. Studies that assessed any duration (acute, subacute, chronic) or frequency (e.g., incidence, prevalence) for a history of LBP were included. This research was the first known attempt to systematically review the literature on functional biomarkers and related characteristics, LBP, and firefighters. Hence, the authors believed that including heterogenous LBP parameters was justified, despite potential limitations of such an approach [31].

Setting: Studies in any setting for which firefighters can perform work activities (actual or simulated), activities of daily living (actual or simulated), or laboratory-based tests were included. Studies could have been conducted in the participant’s natural environment (e.g., work, home, recreational) or in controlled laboratory settings where biomechanical and functional outcomes are measured.

Study types: Full-text peer-reviewed literature of various study types was included, such as cross-sectional, observational cohort, interventional, and controlled trials. Abstracts had to have been available for initial review, and full-text articles had to have been available for final study selection. Studies had to have been original reports of human research. Case reports, review articles, books, government reports, academic theses, dissertations and related publications, non-peer-reviewed publications, and animal, basic science, and laboratory studies were excluded.

### 2.3. Information Sources and Search Strategy

A comprehensive search of PubMed, the Excerpta Medica Database (EMBASE), the Cumulative Index to Nursing and Allied Health Literature (CINAHL), and the Physiotherapy Evidence Database (PEDro) was conducted to identify studies evaluating functional biomarkers in firefighters with or without LBP. The investigators developed search terms for the domains of interest: firefighters, functional biomarkers, and LBP. For LBP, search terms were adapted from previous recommendations of the Cochrane Back and Neck Review Group [33,34]. The PubMed search strategy is shown in Table A2 of Appendix A. Other databases were searched using a comparable approach that was tailored to the database’s specific features. Study publication date was not constrained; databases were searched from their first release through April 2025. Reference lists within eligible studies from the original search, along with investigator files, were also searched.

### 2.4. Selection Process

Covidence systematic review software (https://support.covidence.org/doc/how-can-i-cite-covidence, accessed on 8 October 2025, Veritas Health Innovation, Melbourne, Australia) was used to facilitate the article screening and data extraction processes, including assisting with citation management, screening articles, extracting data, assessing study quality, and synthesizing evidence. Following the searches described above, citations were exported to Covidence software (Melbourne, Australia) and related spreadsheets. Duplicates were removed, and non-English language papers and those without abstracts were excluded. Two authors (JM, RH) independently screened the remaining citations and abstracts to determine initial relevance. The two reviewers discussed disagreements until they reached a consensus. Full text articles were obtained for citations deemed relevant or of uncertain relevance. To establish final eligibility, the two reviewers assessed the full text articles, rectified disagreements, and reached a consensus. Automation instruments were not utilized to select studies.

### 2.5. Data Collection Process

One author (JM) extracted data from the eligible studies and entered these data into spreadsheets and evidence tables. Another author (MB) independently reviewed and verified the extracted data. In addition to the independent and dependent outcome variables described above, other extracted data included demographic variables (e.g., sample size, age, gender, eligibility criteria), study specific variables (e.g., location, funding source), and description of the exposure and intervention. After extracting data, the two reviewers rectified discrepancies until they reached a consensus. If consensus was not reached, a third author (RH) resolved discrepancies. Automation instruments were not utilized to extract data. Missing data are reported in the manuscript, accordingly.

### 2.6. Study Risk of Bias Assessment

The U.S. National Institutes of Health (NIH) instruments for controlled intervention studies and observational cohort and cross-sectional studies were used to assess risk of bias (study quality) [35]. These instruments include 14 items that assess various study characteristics (e.g., methods of randomization, treatment allocation, blinding). Each item is scored as 1 (yes) or 2 (no), and the sum of all items achieves a total score ranging from 0–14. From the total score, study quality categories are 0–4 Poor, 5–9 Fair, and 10–14 Good [27]. The study evidence level was categorized using approaches adapted from the Oxford Centre for Evidence-Based Medicine [27,36,37]. A similar approach with 2–3 authors was used to rate risk of bias and rectify discrepancies, as described for data extraction processes. Automation instruments were not utilized to assess risk of bias, and missing data are reported in the manuscript, accordingly. Examination of reporting bias from missing results was not carried out due to the quantity and quality of available evidence.

Steps were taken to avoid/reduce selection bias in the systematic review process, including the following: key aspects of the study protocol were documented in the registration; multiple databases were searched; all types of studies, publication dates, conference abstracts, years of publication, and grey literature were not excluded from the primary search and were included in the primary search results and subsequent abstract screening (considers unpublished data); and two reviewers independently screened abstracts.

Meta-analysis was not conducted in the review for the reasons described herein, hence publication bias was not assessed through formal statistical analyses such as Egger’s Test or funnel plot asymmetry [38]. Regardless, publication bias was examined through observation of potential differences in outcomes (positive vs. negative) based on study risk of bias and sample size.

### 2.7. Effect Measures

Given the case–control and risk assessment nature of this review, we expected that the analyses within eligible studies would primarily consist of odds ratios, hazard ratios, prevalence ratios, risk ratios, *t*-tests, ANOVA, regression, and similar relational analyses. For all measures, the primary sub-group comparisons were between participants with and without a history of LBP. Meta-analysis and sensitivity analysis were not conducted.

### 2.8. Synthesis Methods

Based on the evidence tables of extracted data for study characteristics, outcomes, type, and quality, two authors (JM, RH) synthesized data, developed empirical evidence statements, and interpreted findings with strategies adapted from the Oxford Centre for Evidence-Based Medicine [39], Clinical Information Access Portal [37], American Physical Therapy Association [19], and three systematic reviews on LBP [26,27,28], along with pertinent features of the Grading of Recommendations Assessment, Development, and Evaluation (GRADE) system [40]. Empirical evidence statements were developed regarding the relationship of LBP in firefighters for each primary dependent outcome variable category described above. Based on risk of bias, study quality, and quantity, each empirical evidence statement was accompanied by an estimate for level of confidence (high, moderate, low, insufficient, confirmatory, conflicting) [26]. The strategy for estimating level of confidence was adapted from a previous systematic review on LBP with a wide array of functional outcome measures, which had a preponderance of evidence from cross-sectional studies [26]. Descriptive criteria for the empirical evidence statements were as follows: Study findings—Support: All comparisons in the study support the empirical evidence statement. Mixed: Some comparisons in the study support the empirical evidence statement, while other comparisons do not support it. Against: All comparisons in the study do not support the empirical evidence statement. Conclusion—Support: ≥Two-thirds of fair–good quality studies support the ESS. Against: ≥Two-thirds of fair–good quality studies do not support the ESS. Inconclusive: Studies do not meet criteria for Support or Against categories. Confidence level—Studies with a high risk of bias were excluded from level-of-confidence estimates. Insufficient Evidence: This includes evidence from ≤1 study of fair–good quality. Low: This includes evidence from 2–3 studies of fair–good quality. Moderate: This includes evidence from 4–5 studies of fair–good quality. High: This includes evidence from ≥6 studies of fair–good quality. All authors reviewed the findings, provided suggestions (as appropriate), and reached a consensus for the final empirical evidence statements.

## 3. Results

### 3.1. Study Selection

**Figure 1:**
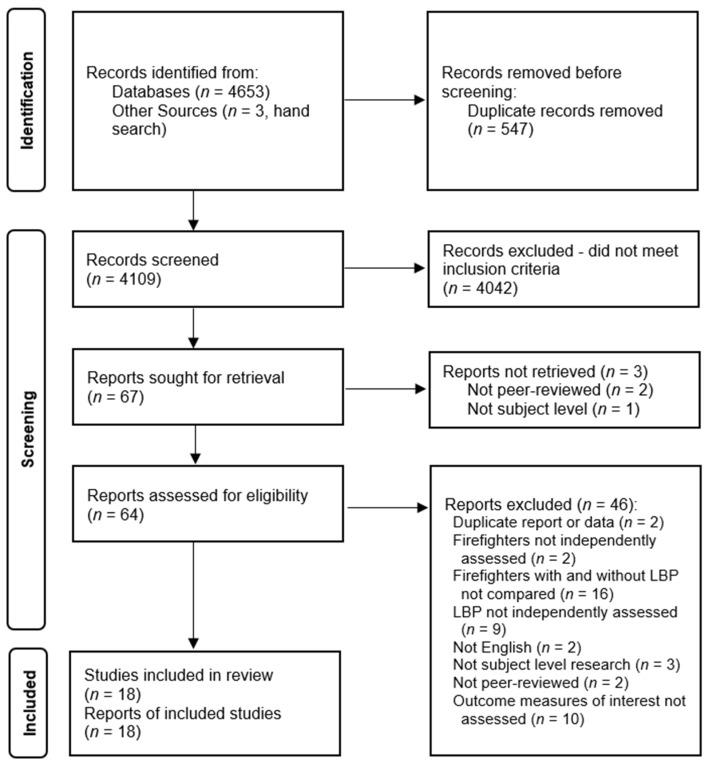
PRISMA diagram of search results.

Search results are reported in the PRISMA diagram, which is shown in Figure 1. Of the 64 full-text reports assessed for eligibility, 18 studies, enrolling 32,977 human subjects, were deemed eligible [6,7,12,41,42,43,44,45,46,47,48,49,50,51,52,53,54,55]. Forty-six reports were excluded for the reasons described in Figure 1.

### 3.2. Study Characteristics and Outcomes

Study characteristics and outcomes are illustrated in Table 1 and Table 2, respectively. Nine studies were conducted in the United States [6,7,12,41,42,50,51,52,55]. The other studies were conducted in China (*n* = 1) [45], Greece (*n* = 1) [46], Korea (*n* = 2) [47,48], Poland (*n* = 1) [43], Singapore (*n* = 1) [49], South Africa (*n* = 2) [53,54], and Spain (*n* = 1) [44]. Ten studies were funded by federal grants [6,7,41,47,48,49,50,51,53,54]. The other studies were funded by a local grant (*n* = 1) [12], private foundation (*n* = 1) [45], private foundation, and federal source (*n* = 1) [44], or had no extramural funding (*n* = 1) [42] and funding not reported (*n* = 4) [43,46,52,55].

Fifteen studies did not report the duration of LBP [6,7,12,41,42,43,46,47,48,49,50,51,52,53,54]. The other studies assessed acute, subacute, and chronic LBP (*n* = 2) [45,55], and chronic LBP (*n* = 1) [44]. Fourteen studies assessed prevalence of LBP [6,7,42,43,44,45,46,47,48,49,50,51,52,55]. The other studies assessed incidence (*n* = 3) [6,12,41], while for some, it was unclear (*n* = 2) [53,54]. Five studies assessed LBP in the past 12 months [46,47,48,49]. The other studies assessed current LBP (*n* = 5) [7,42,43,44,55], unclear time period (*n* = 4) [45,52,53,54], lifetime history of LBP (*n* = 2) [6,55], LBP during a 12-month trial period (*n* = 2) [6,12], back injury in a 4-year period (*n* = 1) [41], back injury in the past 5 years (*n* = 1) [50], and first-time LBP (*n* = 1) [51].

Eight studies assessed anthropometric characteristics [6,7,42,43,45,46,50,53]. The other studies assessed functional work tasks/capacity (*n* = 6) [7,43,46,47,51,54], physical activity (*n* = 6) [7,43,44,46,47,53], muscular fitness (*n* = 5) [6,7,12,49,53], movement quality/range of motion (*n* = 4) [7,12,52,53], disability/kinesiophobia (*n* = 4) [6,7,47,55], kinematics (*n* = 2) [49,52], overall physical fitness (*n* = 2) [41,42], and imaging/structural/morphological characteristics (*n* = 1) [48].

The specific outcome measures used to assess functional biomarkers and related characteristics varied widely and were inconsistently applied among the studies (Table 2). The majority of measures were common tests for which reliability and validity have been established, such as body mass index, waist circumference, physical fitness tests of muscular strength, endurance, and motor control, objective kinematic, motion, and imaging metrics, and self-report measures of kinesiophobia and physical activity. However, other measures were developed by the investigators of each study for which the reliability and validity are unknown, such as aggregate fitness scores and assessment of functional work tasks collected over several time points.

### 3.3. Evidence Level and Risk of Bias

Evidence levels and risks of bias for each study are found in Table 3. Fourteen studies were cross-sectional [7,42,43,44,45,46,47,48,49,51,52,53,54,55], which limits the ability to make causal inferences about LBP risk. The other studies were prospective cohort (*n* = 2) [12,41], randomized controlled trial (*n* = 1) [6], and retrospective cohort (*n* = 1) studies [50]. Two studies were interventional [6,12]. The mean ± SD score for study quality was 7.2 ± 2.0 out of 14, indicating a substantial risk of bias. Thirteen studies were of fair quality (some concerns about risk of bias) [7,12,41,42,47,48,49,50,51,52,53,54,55]. The other studies were of poor quality (high risk of bias) (*n* = 4) [43,44,45,46] and good quality (low risk of bias) (*n* = 1) [6]. When examining study shortcomings illustrated in the 14 items of the risk of bias instrument, it is apparent that most studies lacked details on blinding, power calculation, and confounder control.

No clear signs of publication bias were evident when comparing differences in outcomes (positive vs. negative) based on study risk of bias and sample size. Moreover, there were several occurrences of both positive and negative outcomes being reported in the same study, and many negative outcomes were published in the studies. Thus, study risk of bias and sample size did not appear to influence outcomes or publication.

### 3.4. Results of Syntheses

Empirical evidence statements for each functional biomarker domain and risk of LBP, including study findings, conclusion, and confidence level, are described in Table 4. Overall, the conclusion for most (7/10) of the empirical evidence statements was “Inconclusive” due to mixed findings across the studies. Similarly, the confidence level for most (6/10) of the empirical evidence statements was “Low” or “Insufficient Evidence” due to a lack of studies with fair–good quality from which to draw conclusions. Two of the functional biomarkers (disability/kinesiophobia, functional work tasks/capacity) displayed evidence to support a relationship with risk of LBP at a moderate confidence level, according to the authors’ descriptive grading system. While findings preclude assessment of causal relationships, a higher level of disability/kinesiophobia and poor performance on functional work tasks/capacity were associated with an increased risk of LBP. On the contrary, one functional biomarker (movement quality/range of motion) displayed evidence against a relationship with risk of LBP, with a low confidence level. Two interventional studies supported the clinical benefit of exercise interventions targeting trunk muscular fitness alone [6], or when trunk muscular fitness exercises are combined with movement quality/range-of-motion exercises [12].

## 4. Discussion

The findings of this systematic review highlight the critical burden of LBP among firefighters, as well as limitations of the existing evidence base. Across 18 studies enrolling nearly 33,000 participants, we observed substantial heterogeneity in study design, functional biomarker domains, and outcome measures, with most studies being cross-sectional and of only fair methodological quality. The vast majority of empirical evidence statements (7 of 10) were rated as inconclusive, and confidence levels for six domains were low or insufficient, underscoring the lack of definitive guidance for clinical or occupational applications. Two functional biomarkers, disability/kinesiophobia and performance on functional work tasks, emerged as consistent predictors of LBP, with moderate confidence in the evidence according to the authors’ descriptive grading system. Conversely, other domains such as anthropometrics, muscular fitness, and physical activity, while well established in the general population, showed inconsistent or inconclusive associations in firefighters.

Key findings of this review include the following: 1. Numerous knowledge gaps exist in the peer-reviewed literature about the relationships among functional biomarkers and the risk of LBP in firefighters. Given the overall weakness and fragmentation of the evidence, robust claims about the potential clinical implications of functional biomarkers are likely premature. 2. Several studies, which were excluded from analysis, assessed functional measures in healthy firefighters or those with general musculoskeletal disorders and injuries, but did not separately assess the effects of LBP. 3. The outcomes assessed varied widely across the studies, such as different definitions of LBP and heterogenous functional biomarkers, which limits the ability to make clinical interpretations and precludes meta-analysis. 4. Most of the included studies (14/18) were cross-sectional, which limits the ability to make causal inferences about LBP risk. Only one study was rated as good-quality, and the average quality score was 7.2/.14, which suggests a substantial risk of bias overall. 5. Few studies considered the impact of multiple functional biomarkers together (e.g., covariates, regression). 6. Findings, as depicted in the empirical evidence statements, were mainly mixed and inconclusive. Some studies showed a substantial relationship between LBP and the assessed functional biomarker, while others showed no relationship. 7. Only monotherapies (e.g., exercise) aimed at closely aligned functional biomarkers were assessed in the two interventional studies, and only one of the interventional studies showed low risk of bias. 8. None of the eligible studies compared firefighters with and without LBP using gold standard biomechanical assessments for the spine.

Limitations of the evidence uncovered in this review should be acknowledged. First, several studies in the available literature exhibited fair-to-poor methodological quality (some concerns about bias or high risk of bias) and had cross-sectional designs, which limit the ability to infer causality or temporal relationships between functional biomarkers and LBP in firefighters. Second, the substantial heterogeneity in study populations, exposure definitions, outcome measures, and functional assessment methods precluded meta-analysis and reduced comparability across studies. Third, many studies lacked detailed reporting on LBP chronicity, occupational exposure, or firefighter-specific work demands, restricting generalizability to the broader tactical athlete population. Fourth, advanced biomechanical, neuromuscular, and imaging assessments were rarely incorporated, resulting in incomplete characterization of the multidimensional risk factors that are likely to contribute to LBP. Fifth, a standardized definition of functional biomarkers for LBP in high-risk occupations does not exist. Thus, the operational definition of functional biomarkers for this review was arbitrarily selected by the authors and, by design, did not integrate all components of the biopsychosocial model. Finally, the fragmentation of evidence, as described herein, has precluded a holistic understanding of the complex biopsychosocial mechanisms underlying LBP in firefighters. This gap represents a critical barrier to the development of targeted, evidence-based interventions for prevention, treatment, and workforce readiness.

Since the limitations of the evidence uncovered in this review preclude generalizability and widespread implementation within the fire service, confirmatory research is needed to guide clinical decision-making. There is an urgent need for rigorous, prospective, multimodal studies to establish mechanistic clarity, examine standardized theoretical models for functional domains, validate predictive biomarkers, and inform evidence-based prevention and rehabilitation strategies in firefighters. Future research must move beyond fragmented, single-domain studies and adopt integrative, prospective designs that capture the full spectrum of biomechanical, neuromuscular, functional, and biopsychosocial determinants of LBP in firefighters. In this population, research is needed to assess the validity and implementation of advanced motion analysis technologies, including wearable inertial measurement units (IMUs) and markerless motion capture, to assess the validity of such technologies to monitor functional performance during occupational and training activities. Likewise, the validity of integrating measures of anthropometric characteristics, muscular fitness, imaging metrics, disability/kinesiophobia, and task-specific performance (e.g., lifting, carrying, and climbing), along with other biopsychosocial domains, needs to be assessed. Interventional research should extend beyond physical fitness exercises to integrated programs that address various elements, such as strength, endurance, motor control, functional patterns, anthropometric characteristics, and biopsychosocial barriers, such as kinesiophobia [7].

From a clinical perspective, the available evidence prevents making robust and broad recommendations about pragmatic applications for functional biomarkers for occupational health in the fire service. Nonetheless, some recommendations can be made for the functional biomarkers with a moderate level of evidence according to the authors’ descriptive grading system. For example, disability and kinesiophobia are highly actionable domains. Early identification of “yellow flags” may help identify firefighters at elevated risk of recurrent or chronic LBP and guide targeted rehabilitative interventions that integrate behavioral and physical approaches, such as cognitive behavioral therapy and trunk muscular fitness exercises. Similarly, poor performance on work-related tasks is particularly relevant, as the physical demands of firefighting appear to require optimal biomechanical efficiency under high load and stress. Addressing deficits in work-related tasks is feasible through goal-oriented plans of care focusing on objective measures of physical performance and work-related activities that are tailored to the individual’s needs. Details of such programs are beyond the scope of the available evidence from this review. However, the authors recommend that clinicians implement specific strategies for decision-making according to the foundations of evidence-based practices—research evidence, clinical expertise, and patient values [56].

In addition to the aforementioned considerations for clinical decision-making, the current synthesis highlights the need to assess and adopt advanced conceptual and analytical frameworks to better characterize the complex, nonlinear dynamics underlying spinal function and LBP in firefighters. The limited evidence for validated task-specific functional biomarkers underscores the inadequacy of conventional linear models in fully capturing adaptive and maladaptive physiological responses. Increasing evidence suggests that disrupted coordination between automatic and voluntary neuromuscular systems may reflect protective movement strategies that, if persistent, evolve into maladaptive motor control patterns detectable through nonlinear analyses of movement variability and motor coordination [57,58]. Collectively, these findings may align with the inherently nonlinear nature of LBP [59], suggesting that fluctuations in pain, motor control, and recovery trajectories may represent dynamic adaptations rather than isolated biomechanical deficits, which could ultimately influence clinical decision-making and rehabilitation planning. Variability in recovery and return-to-duty outcomes may therefore represent inherent nonlinear system behavior rather than methodological inconsistency [60]. While these concepts are beyond the scope of the evidence synthesized in this review, they provide direction for future research to elucidate the clinical implications of nonlinear system behavior in LBP, specifically related to functional biomarkers in firefighters.

## 5. Conclusions

Firefighters experience uniquely high exposures to spinal loading, hazardous work environments, irregular schedules, and other occupational stressors, positioning them at elevated risk for LBP and its associated functional disabilities. This systematic review highlights the substantial knowledge gaps in the understanding of functional biomarkers related to LPB in this tactical athlete population. While select studies support associations among disability/kinesiophobia, functional work task performance, and LBP, the overall evidence base is fragmented, heterogeneous, and largely inconclusive. The limited interventional evidence, focusing primarily on trunk muscular fitness, demonstrates the benefit of addressing modifiable risk factors, but underscores the urgent need for more comprehensive approaches.

Collectively, the findings of this review preclude mechanistic understanding, predictive modeling, and precise recommendations for evidence-based prevention and rehabilitation strategies for this workforce. Future clinical trials and implementation research efforts are needed to examine multimodal assessments and interventions, incorporating advanced biomechanics, functional performance, anthropometric characteristics, physical fitness, imaging, and psychosocial profiling within tailored and patient-centered approaches. If positive findings are found in this research, such integrative efforts have the potential to identify modifiable risk factors, optimize injury prevention and recovery strategies, and inform return-to-work protocols across the continuum of prevention, clinical care, and resilience development. By systematically bridging the gap between functional biomarkers and clinical outcomes, these efforts can help transform the management of LBP, reduce disability, enhance recovery and operational readiness, and provide a scalable model for spine health initiatives in firefighters and other tactical athletes.

## Figures and Tables

**Table 1:** Study characteristics.

Author, Year, Location, Funding Source	Population, *n* (Gender), Age	Eligibility Criteria	Low Back Pain: Chronicity. Prevalence/Incidence. Time Period	Functional Domain
Cady et al., 1979 [41], USA, Federal Grant	Firefighters from a municipal department, 1652 (NR), 20–55 y	Inclusion: firefighter from collaborating department, age 20–55 y. Exclusion: not fully recovered from back injury, back injury related to MVA. No prior unresolved back injuries or orthopedic conditions interfering with strength measurements, abnormal exercise electrocardiograms.	NR. Incidence. Back injury in 4-year period	9
Damrongsak et al., 2018 [42], USA, No extramural funding	Career firefighters from a municipal department, 298 (0 F, 298 M), 39.6 ± 9.8 y	Inclusion: career firefighter from collaborating department, full time, age 20–60 y, male. Exclusion: spinal fracture or malignancy.	NR. Prevalence. Current LBP	2, 9
Fiodorenko-Dumas et al., 2018 [43], Poland, NR	Firefighters, 61 (1 F, 60 M), 33.8 (20–56) y	Inclusion: working in firefighting service ≥ 1 y. Exclusion: spinal fracture, mechanical spine injury, spinal or prevertebral muscle malignancy or infection, neurological disease associated with spinal pain.	NR. Prevalence. Current LBP	2, 4, 10
García-Heras et al., 2022 [44], Spain, Private Foundation, Federal Source	Wildland firefighters, 223 (18 F, 203 M), 36.4 ± 6.3 y	Inclusion: wildland firefighters from Spanish Forest Fire Reinforcement Brigades. Exclusion: NR.	Chronic. Prevalence. Current LBP	10
Gong et al., 2023 [45], China, Private Foundation	Firefighters from various municipal districts, 214 (0 F, 214 M), 29.4 ± 2.7 y	Inclusion: firefighter from collaborating district, male, age 20–40 y, good physical health. Exclusion: underlying disease, MSD history before firefighting career.	Acute, Subacute, Chronic. Prevalence. Unclear	2
Katsavouni et al., 2014 [46], Greece, NR	Career firefighters, 3451 (124 F, 3327 M), 38.1 ± 7.3 y	Inclusion: career firefighter. Exclusion: volunteer firefighter.	NR. Prevalence. LBP in past 12 months	2, 4, 10
Kim et al., 2017 [47], Korea, Federal Grant	Firefighters, 24,209 (0 F, 24,209 M), 30–39 y (median)	Inclusion: firefighters from South Korea, male. Exclusion: NR.	NR. Prevalence. LBP in past 12 months	3, 4, 10
Kim et al., 2021 [48], Korea, Federal Grant	Career firefighters, 297 (0 F, 297 M), 30–39 y (median)	Inclusion: firefighters from South Korea, male. Exclusion: NR.	NR. Prevalence. LBP in past 12 months	5
Kong et al., 2024 [49], Singapore, Federal Grant	Career firefighters, 42 (0 F, 42 M), 31.5 ± 5.1 y	Inclusion: career full active-duty frontline firefighter from Singapore Civil Defence Force, age 21–45 y. Exclusion: history of back surgery, pregnant.	NR. Prevalence. LBP in past 12 months	6, 8
Kuehl et al., 2012 [50], USA, Federal Grant	Firefighters enrolled in PHLAME study, 433 (22 F, 411 M), 39.2 y	Inclusion: firefighters enrolled in wellness program from collaborating department. Exclusion: NR.	NR. Prevalence. Back injury in past 5 years	2
Mayer et al., 2020 [6], USA, Federal Grant	Career firefighters from 4 municipal departments, 264 (32 F, 232 M), 35.1 ± 8.6 y	Inclusion: career firefighter, full active duty, without work restrictions, in regular service, assigned to fire station from a collaborating department. Exclusion: current workers’ compensation or personal injury claim, pregnant.	NR. Prevalence, Incidence. Lifetime history of LBP, LBP during 12-month trial period	2, 3, 8
Mayer et al., 2024 [7], USA, Federal Grant	Career firefighters from 15 municipal departments, fire protection districts, and reservation departments, 419 (5 F, 414 M), 37.6 ± 8.8 y	Inclusion: career firefighter from collaborating department, full duty, without work restrictions. Exclusion: relevant current workers’ compensation or personal injury claim in past 30 days or that has not reached MMI, pregnant.	NR. Prevalence. Current LBP	2, 3, 4, 7, 8, 10
Nuwayhid et al., 1993 [51], USA, Federal Grant	Career firefighters from a municipal department, 224 (NR), NR	Inclusion: career firefighter, first-time LBP with seeking care or with lost work time (case). Exclusion: LBP associated with internal diseases, e.g., urinary tract infection, malignancy.	NR. Prevalence. First-time LBP	4
Parkhurst et al., 1994 [52], USA, NR	Firefighters from municipal departments, 88 (0 F, 88 M), 32.7 ± 8.1 y	Inclusion: firefighter, active duty. Exclusion: acute pain, receiving treatment for back injury.	NR. Prevalence. Unclear	6, 7
Peate et al., 2007 [12], USA, Local Grant	Career firefighters from a municipal department, 433 (25 F, 408 M), 41.5 y	Inclusion: career full active-duty firefighter. Exclusion: NR.	NR. Incidence. LBP during 12-month study period	4, 7, 8
Ras et al., 2023 [53], South Africa, Federal Grant	Career firefighters from a municipal department, 308 (34 F, 274 M), 20–65 y	Inclusion: career active-duty firefighter, age 20–65 y. Exclusion: administrative duty, part-time or seasonal duty, sick leave.	NR. Unclear. Unclear	1, 2, 7, 8, 10
Ras et al., 2024 [54], South Africa, Federal Grant	Career firefighters from a municipal department, 279 (31 F, 248 M), 20–65 y	Inclusion: career active-duty firefighter, age 20–65 y. Exclusion: administrative duty, part-time or seasonal duty, sick leave.	NR. Unclear. Unclear	1, 4
Stassin et al., 2021 [55], USA, NR	Career firefighters from a municipal department, 82 (3 F, 79 M), 41 ± 9 y	Inclusion: career firefighter from collaborating department. Exclusion: NR.	Acute, subacute, and chronic. Prevalence. Lifetime history of LBP. Current LBP	3

Key: F: female; LBP: low back pain; M: male; MMI: Maximum Medical Improvement; MSD: musculoskeletal disorder; MVA: Motor Vehicle Accident; NR: Not Reported; Y: Year. Functional domain—1: aerobic capacity; 2: anthropometric measures; 3: disability/kinesiophobia; 4: functional work tasks/capacity; 5: imaging/structural/morphological characteristics; 6: kinematics; 7: movement quality/range of motion; 8: muscular fitness; 9: overall physical fitness; and 10: physical activity.

**Table 2:** Study characteristics.

Author, Year	Outcome Measure: Low Back Pain	Outcome Measure: Functional Biomarker	Case/Intervention. Control	Analysis. Results
Cady et al., 1979 [41]	Back injury frequency: Yes/No (workers’ compensation administrative data).	Overall physical fitness: aggregate score derived from physical fitness tests—aerobic capacity, isometric muscular strength, and spinal flexibility. Grouped by fitness level (most fit, middle fit, and least fit).	Case: Highest fitness (*n* = 259). Fitness and conditioning program. Control: Middle fitness (*n* = 1127), lowest fitness (*n* = 266). Fitness and conditioning program.	Regression. Participants with the highest level of baseline physical fitness had a lower risk of subsequent work-related back injury (highest: 0.7%; middle 3.2%; lowest 7.1%; *p* < 0.05).
Damrongsak et al., 2018 [42]	Current LBP: Yes/No (PRO).	Anthropometric measures: BMI (kg/m^2^). Overall physical fitness: aggregate score from 1.5-mile run or 3-mile walk, sit-and-reach flexibility, 1-min sit-ups in 1 min, and bench press 1-RM strength.	Case: With current LBP (*n* = 90). Control: Without current LBP (*n* = 208).	Regression. NS: No difference in BMI between participants with and without current LBP. NS: Aggregate physical fitness score did not add to prediction of current LBP from regression model with variables of age, LBP lifetime history, BMI, job satisfaction, job stress, and peer support.
Fiodorenko-Dumas et al., 2018 [43]	LBP frequency: NRS (0–10, PRO).	Anthropometric measures: BMI (kg/m^2^). Functional work tasks/capacity: application of ergonomic principles for standing, sitting, and lifting heavy objects (survey, PRO). Physical activity: IPAQ (PRO).	Case: NA (1-arm observational study). Control: NA (1-arm observational study).	Pearson product–moment correlation coefficient. LBP frequency was associated with BMI (r = 0.324, *p* = 0.01) and application of ergonomic principles (r = 0.263, *p* = 0.02). NS: Physical activity during day, at work, and in leisure time.
García-Heras et al., 2022 [44]	Current chronic LBP: Yes/No (PRO).	Physical activity: leisure time exercise training (hr/wk, PRO). Leisure time preventive training (Yes/No, PRO).	Case: With current chronic LBP (*n* = 70). Control: Without current chronic LBP (*n* = 153).	Chi-square. NS: No difference in leisure time exercise training and leisure time preventive training between participants with and without current chronic LBP.
Gong et al., 2023 [45]	Training-related back injury: Yes/No (PRO).	Anthropometric measures: BMI (kg/m^2^, self-reported, PRO).	Case: With training-related back injury (NR). Control: Without training-related back injury (NR).	Regression. NS: No difference in self-reported BMI between those with and without training-related back injury.
Katsavouni et al., 2014 [46]	LBP in past 12 months: Yes/No (PRO).	Anthropometric measures: BMI (kg/m^2^, self-reported, PRO). Functional work tasks/capacity: lifting load at work (kg, self-reported, PRO). Physical activity: exercise frequency (hr/wk, PRO).	Case: With LBP in past 12 months (*n* = 1037). Control: Without LBP in past 12 months (*n* = 2414).	Regression. Risk of LBP in the past 12 months is lower in those who exercise (1–5 hr/wk or >5 hr/wk) compared with no exercise (1–5 hr/wk OR 0.49 (95% CI 0.41–0.58); >5 hr/wk OR 0.41 (95% CI 0.31–0.53); *p* < 0.01). Risk of LBP in the past 12 months is higher in those who self-reported lifting > 10 kg and >25 kg compared with no lifting (>10 kg OR 1.62 (95% CI 1.35–1.94); >25 kg OR 1.50 (95% CI 1.29–1.74); *p* < 0.01). NS: No difference in self-reported BMI between those with and without LBP in the past 12 months.
Kim et al., 2017 [47]	LBP in past 12 months: Yes/No (PRO).	Disability/kinesiophobia: Korean Occupational Stress Scale (KOSS) sub-scales of uncomfortable physical environment, high job demand, and discomfort in occupational climate. Functional work tasks/capacity: heavy lifting load at work (categories, PRO). Awkward posture at work (categories, PRO). Physical activity: exercise frequency (d/wk, PRO).	Case: With LBP in past 12 months (*n* = 4671). Control: Without LBP in past 12 months (*n* = 19,538).	Regression. A higher percentage of participants with LBP in the past 12 months reported the worst level on KOSS sub-scales of uncomfortable physical environment (with 69.8%; without 46.0%; *p* < 0.05), high job demand (with 55.4%; without 37.7%; *p* < 0.05), and discomfort in occupational climate (with 57.4%; without 46.1%; *p* < 0.05). A higher percentage of participants with LBP in the past 12 months reported heavy lifting at work for most of day (with 28.1%; without 11.6%; *p* < 0.05) and awkward work posture for most of day (with 23.1%; without 11.4%; *p* < 0.05). NS: No difference in exercise frequency between those with and without LBP in the past 12 months.
Kim et al., 2021 [48]	LBP in past 12 months: Yes/No (PRO).	Imaging/structural/morphological characteristics: L4-L5 disc herniation and L5-S1 central canal stenosis (MRI).	Case: With LBP in past 12 months (*n* = 213). Control: Without LBP in past 12 months (*n* = 84).	Regression. Participants with LBP in the past 12 months had a higher risk of L4-L5 disc herniation than those without LBP in the past 12 months (OR 1.86, 95% CI 1.03–3.35, *p* < 0.05). NS: No difference in L5-S1 central canal stenosis between those with and without LBP in the past 12 months.
Kong et al., 2024 [49]	LBP in past 12 months: Yes/No (PRO).	Kinematics: passive muscle stiffness—L1 longissimus (myotonometry, Nm). Trunk extension muscle fatigability—L1 longissimus (EMG). Muscular fitness: Isometric Back Extension Strength (dynamometer, kg).	Case: With LBP in past 12 months (*n* = 23). Control: Without LBP in past 12 months (*n* = 19).	*t*-test. NS: No difference in passive muscle stiffness, trunk extension muscle fatigability, and isometric trunk extension strength between those with and without LBP in the past 12 months.
Kuehl et al., 2012 [50]	Back injury in past 5 years: Yes/No (PRO associated workers’ compensation claims).	Anthropometric measures: BMI (kg/m^2^).	Case: With back injury over past 5 years (*n* = 60). Control: Without back injury over past 5 years (*n* = 333).	Chi-square. NS: No difference in BMI categories (normal, overweight, obese) between participants with and without back injuries over the past 5 years.
Mayer et al., 2020 [6]	Lifetime history of LBP: Yes/No (PRO). LWT (hours) associated with LBP over 12-month trial period.	Anthropometric measures: BMI (kg/m^2^). Disability: BBQ (9–45, PRO). Muscular fitness: back and core muscular endurance—BST, PPT (sec).	Intervention (*n* = 181): Back and core muscle exercise program: 2x/wk, 12 mo, supervised (*n* = 86) or telehealth (*n* = 95) delivery. Control (*n* = 83): Brief education: 1X.	Regression, ANOVA, *t*-test. Exercise reduced LWT related to LBP over the12-month trial period. For each hour of LWT in supervised exercise group, control group had 1.15 h (95% CI: 1.04–1.27; *p* = 0.008). For each hour of LWT in telehealth exercise group, control group had 5.51 h (95% CI: 4.53–6.70; *p* < 0.0001), and supervised exercise group had 4.8 h (95% CI: 3.9–5.9; *p* < 0.0001). Participants with a history of LBP had worse baseline BMI (with 29.0 ± 4.3; without: 27.7 ± 3.8; *p* = 0.003) and BBQ (with 28.4 ± 5.7; without 27.7 ± 6.0; *p* = 0.006); NS: BST, PPT. Participants who experienced LBP during the 12-month trial period had worse baseline BMI (with 29.0 ± 3.8; without 27.4 ± 3.8; *p* = 0.01) and BST (with 68.0 ± 30.0; without 78.3 ± 32.0; *p* = 0.009); NS: BBQ, PPT.
Mayer et al., 2024 [7]	Current LBP: Yes/No (PRO).	Anthropometric measures: BMI (kg/m^2^), bodyfat (%) and waist circumference (cm). Disability/kinesiophobia: ODI (0–100%, PRO), perceived disability (% participants, PRO), FAFQ (0–20, PRO). Functional work tasks/capacity: FFTQ (0–48, PRO), MTAP-LC (0–56, PRO), and perceived PDC (% participants, PRO). Movement quality: FMS-4 sub-tests—squat, hurdle, lunge, and SLR (0–12). Muscular fitness: back and core muscular endurance—BST, PPT (sec). Physical activity: EF—strength and cardio flexibility (d/wk, PRO).	Case: With current LBP (*n* = 83). Control: Without current LBP (*n* = 336).	ANOVA. Participants with current LBP had worse BMI (with 29.3 ± 4.0; without 28.3 ± 3.6; *p* = 0.031), bodyfat (with 23.6 ± 6.0; without 21.3 ± 5.9; *p* = 0.002), waist circumference (with 96.4 ± 11.2; without 93.1 ± 9.6; *p* = 0.007), ODI (with 10.2 ± 7.5; without 2.8 ± 4.1; *p* < 0.001), perceived disability ≥ minimal per ODI (with 96.4%; without 51.8%; *p* < 0.001), FAFQ (with 3.6 ± 3.1; without 2.2 ± 2.1; *p* < 0.001), FFTQ (with 39.9 ± 5.8; without 41.7 ± 5.8; *p* = 0.012), MTAP-LC (with 53.9 ± 3.2; without 54.9 ± 2.7; *p* = 0.038), perceived PDC below very heavy job demands per MTAP-LC (with 51.3%; without 34.3%; *p* = 0.044), BST (with 179.6 ± 104.8; without 206.4 ± 100.7; *p* = 0.034), EF—strength (with 2.4 ± 1.6; without 3.1 ± 1.6; *p* = 0.001), EF—cardio (with 2.7 ± 1.5; without 3.4 ± 1.4; *p* < 0.001), and EF—flexibility (with 1.9 ± 1.6; without 2.4 ± 1.7; *p* = 0.006). NS: FMS-4, PPT.
Nuwayhid et al., 1993 [51]	First-time LBP: Yes/No (administrative data).	Functional Work Tasks/Capacity: Work activities performed at last work shift before LBP occurrence (PRO).	Case: With first-time LBP (*n* = 115). Control: Without first-time LBP (*n* = 109).	Regression. Work activities performed at last shift that were associated with high risk of first-time LBP include cutting structures OR 6.47 (95% CI 2.05–20.46), breaking windows OR 4.45 (95% CI 1.90–10.42), looking for hidden fires OR 4.32 (95% CI 1.66–11.29), operating charged hose inside building OR 3.26(95% CI 1.32–8.02), climbing ladders OR 3.18 (95% CI 1.07–9.50), and lifting objects ≥ 18 kg OR 3.07 (95% CI 1.29–7.88).
Parkhurst et al., 1994 [52]	History of low back injury: Yes/No (NR).	Kinematics: lumbar proprioception—various raw measures and derived variables from spinal motion device, e.g., passive motion threshold, directional motion perception, and repositioning accuracy. Movement quality/ROM: lumbar flexion ROM (Schober Test).	Case: With history of low back injury (*n* = 33). Control: Without history of low back injury (*n* = 55).	Correlation and regression. History of low back injury was related to coronal (r = 0.22, *p* ≤ 0.05) and sagittal (r = 0.17, *p* ≤ 0.05) passive motion threshold asymmetry, and lumbar flexion ROM (r = −0.26, *p* ≤ 0.05). NS: No relationship between history of low back injury and transverse passive motion threshold asymmetry, and the 12 raw proprioception measures.
Peate et al., 2007 [12]	Back injuries over 12-month study period: total (#) and those with LWT (#).	None	Intervention (*n* = 433): education and exercise training focusing on trunk strength, endurance, motor control, flexibility, movement quality, and body mechanics, with 21 sessions, over 2 months. Control (NR): historical control derived from previous 12 months, details not provided.	Two-sample test of proportion. Compared to historical controls, the intervention group experienced a 44% reduction in number of back injuries (intervention 22; control 39; *p* = 0.024) and 62% reduction in number of back injuries resulting in LWT (intervention 11; control 29; *p* = 0.004) during the 12-month study period.
Ras et al., 2023 [53]	LBP: Yes/No (PRO). Low back injury: Yes/No (PRO).	Aerobic capacity: VO2 predicted. Anthropometric measures: Lean body mass: BIA (%). Movement quality/ROM: Sit-and-Reach test (cm). Muscular fitness: Grip strength (kg), leg strength (kg), push-ups (RPM), and sit-ups (RPM). Physical activity: IPAQ (PRO).	Case: With LBP (*n* = 68–71). With low back injury (*n* = 24). Control: Without LBP (*n* = 235–238). Without low back injury (*n* = 279–285).	ANOVA, *t*-test, and regression. Participants with LBP had worse VO2 rel (*p* = 0.030) and greater amount of lower intensity physical activity (*p* = 0.002). NS: VO2 abs, grip strength, leg strength, push-ups, sit-ups, sit-and-reach, lean body mass. Participants with low back injuries had worse push-ups (*p* = 0.036). NS: VO2 abs, VO2 rel, grip strength, leg strength, sit-ups, sit-and-reach, lean body mass, and physical activity.
Ras et al., 2024 [54]	LBP: Yes/No (PRO). Low back injury: Yes/No (PRO).	Functional work tasks/capacity: Firefighter Physical Ability Test (time- and form-based)step-up, charged hose drag and pull, forcible entry, equipment carry, ladder raise and extension, and rescue drag.	Case: With LBP (*n* = 56–59). With low back injury (*n* = 20–22). Control: Without LBP (*n* = 211–220). Without low back injury (*n* = 247–257).	Mann–Whitney U, Kruskal–Wallis H, and regression. Participants with LBP had worse ladder raises and extensions (*p* = 0.046). NS: step-up, charged hose drag and pull, forcible entry, equipment carry, and rescue drag. Participants with low back injury had worse step-up (*p* = 0.010), charged hose drag and pull (*p* = 0.013), ladder raise, and extension results (*p* = 0.027). NS: forcible entry, equipment carry, and rescue drag.
Stassin et al., 2021 [55]	Lifetime history of LBP: Yes/No (PRO). Current LBP: Yes/No (PRO). Duration of current LBP: acute, subacute, and chronic (PRO).	Disability/kinesiophobia: FABQ (0–96, PRO).	Case: With lifetime history LBP (*n* = 77). With current LBP (*n* = 36). Duration of current LBP: chronic (*n* = 28) and acute (*n* = 6). Control: Without lifetime history LBP (*n* = 5). Without current LBP (*n* = 41). Duration of current LBP: subacute (*n* = 2).	Effect sizes. Participants with history of LBP had worse FABQ (with 22.3 ± 14.5; without 0 ± 0; η^2^ = 0.31). Participants with current LBP had worse FABQ (with 30.8 ± 11.6; without 14.7 ± 12.6; η2 = 0.13). Participants with acute or chronic LBP had worse FABQ than subacute LBP (acute 32.2 ± 9.5, subacute 20.0 ± 2.8; chronic 31.2 ± 12.1; η2 = 0.05).

Key: #: Number; ANOVA: Analysis of Variance; BBQ: Back Beliefs Questionnaire; BMI: body mass index; BST: Biering–Sorensen Test (modified); CI: Confidence Interval; EF: exercise frequency; FABQ: Fear Avoidance Behavior Questionnaire; FAFQ: Fear and Fatigue Questionnaire; FMS: Functional Movement Screen; hr: hour; IPAQ: International Physical Activity Questionnaire; LBP: low back pain; LWT: lost work time; mo: month; MRI: Magnetic Resonance Imaging; MTAP-LC: Multidimensional Task Ability Profile—Lift and Carry sub-test; NR: Not Reported; NRS: Numerical Rating Scale; NS: Not Significant; ODI: Oswestry Disability Index; OR: odds ratio; PDC: Physical Demand Characteristics of Work Level; PPT: Prone Plank Test; PRO: patient-reported outcome; RM: Repetition Maximum; ROM: range of motion; RPM: Repetition Per Minute; SLR: straight leg raise; VO2: Oxygen Uptake (Aerobic Capacity); wk: week.

**Table 3:** Study evidence level and quality (risk of bias).

		Study Quality (Risk of Bias)																
		Item Number																
Author, Year	Evidence Level, Study Type	1	2	3	4	5	6	7	8	9	10	11	12	13	14	Total Score	Study Quality	Risk of Bias
***Observational Cohort and Cross-Sectional Studies:***																		
Cady et al., 1979 [41]	2. Prospective Cohort	Y	Y	NR	Y	N	Y	Y	N	Y	Y	Y	NR	NR	N	8	Fair	**Some**
Damrongsak et al., 2018 [42]	4. Cross-Sectional	Y	Y	NR	Y	Y	NR	Y	N	Y	N	Y	NR	NA	Y	8	Fair	**Some**
Fiodorenko-Dumas et al., 2018 [43]	4. Cross-Sectional	Y	N	NR	Y	N	NR	NR	NR	Y	N	Y	NR	NA	N	4	Poor	**High**
García-Heras et al., 2022 [44]	4. Cross-Sectional	Y	Y	N	Y	N	NA	Y	N	Y	N	Y	NA	NA	N	5	Poor	**High**
Gong et al., 2023 [45]	4. Cross-Sectional	Y	Y	Y	Y	N	NA	Y	N	N	N	N	NA	NA	N	5	Poor	**High**
Katsavouni et al., 2014 [46]	4. Cross-Sectional	Y	Y	NR	Y	N	NA	Y	N	Y	N	N	NA	NA	N	5	Poor	**High**
Kim et al., 2017 [47]	4. Cross-Sectional	Y	Y	Y	Y	N	NA	Y	N	Y	N	Y	NA	NA	N	7	Fair	**Some**
Kim et al., 2021 [48]	4. Cross-Sectional	Y	Y	NR	Y	N	Y	Y	N	Y	N	Y	NR	NA	Y	8	Fair	**Some**
Kong et al., 2024 [49]	4. Cross-Sectional	Y	Y	NR	Y	N	Y	Y	N	Y	N	Y	NR	NA	N	7	Fair	**Some**
Kuehl et al., 2012 [50]	3. Retrospective Cohort	Y	Y	NR	Y	N	Y	Y	Y	Y	Y	Y	NR	NR	N	9	Fair	**Some**
Mayer et al., 2024 [7]	4. Cross-Sectional	Y	Y	Y	Y	Y	Y	Y	N	Y	N	Y	N	NA	N	9	Fair	**Some**
Nuwayhid et al., 1993 [51]	4. Cross-Sectional	Y	Y	NR	Y	N	Y	Y	N	Y	N	Y	NR	NA	Y	8	Fair	**Some**
Parkhurst et al., 1994 [52]	4. Cross-Sectional	Y	Y	NR	Y	N	NR	Y	N	N	N	Y	NR	NA	N	5	Fair	**Some**
Peate et al., 2007 [12]	2. Prospective Cohort	Y	Y	NR	Y	N	Y	Y	Y	Y	Y	Y	NR	NR	N	9	Fair	**Some**
Ras et al., 2023 [53]	4. Cross-Sectional	Y	Y	NR	Y	N	NR	Y	N	N	N	Y	NR	NA	Y	6	Fair	**Some**
Ras et al., 2024 [54]	4. Cross-Sectional	Y	Y	NR	Y	N	NR	Y	N	N	N	Y	NR	NA	Y	6	Fair	**Some**
Stassin et al., 2021 [55]	4. Cross-Sectional	Y	Y	NR	Y	N	Y	Y	Y	Y	N	Y	NA	NR	N	8	Fair	**Some**
***Randomized Controlled Trials (RCTs):***																		
Mayer et al., 2020 [6]	2. RCT	Y	Y	Y	N	Y	Y	Y	Y	N	Y	Y	Y	Y	Y	12	Good	**Low**

Key: Item number: From the NIH quality assessment tool [30]. ***For observational cohort and cross-sectional studies***: 1. Was the research question or objective in this paper clearly stated? 2. Was the study population clearly specified and defined? 3. Was the participation rate of eligible persons at least 50%? 4. Were all the subjects selected or recruited from the same or similar populations (including the same time period)? Were inclusion and exclusion criteria for being in the study prespecified and applied uniformly to all participants? 5. Was a sample size justification, power description, or variance and effect estimates provided? 6. For the analyses in this paper, were the exposure(s) of interest measured prior to the outcome(s) being measured? 7. Was the timeframe sufficient so that one could reasonably expect to see an association between exposure and outcome if it existed? 8. For exposures that can vary in amount or level, did the study examine different levels of the exposure as related to the outcome (e.g., categories of exposure, or exposure measured as continuous variable)? 9. Were the exposure measures (independent variables) clearly defined, valid, reliable, and implemented consistently across all study participants? 10. Was the exposure(s) assessed more than once over time? 11. Were the outcome measures (dependent variables) clearly defined, valid, reliable, and implemented consistently across all study participants? 12. Were the outcome assessors blinded to the exposure status of participants? 13. Was loss to follow-up after baseline 20% or less? 14. Were key potential confounding variables measured and adjusted statistically for their impact on the relationship between exposure(s) and outcome(s)? ***For controlled intervention studies (randomized controlled trials):*** 1. Was the study described as randomized, a randomized trial, a randomized clinical trial, or an RCT? 2. Was the method of randomization adequate (i.e., use of randomly generated assignment)? 3. Was the treatment allocation concealed (so that assignments could not be predicted)? 4. Were study participants and providers blinded to treatment group assignment? 5. Were the people assessing the outcomes blinded to the participants’ group assignments? 6. Were the groups similar at baseline on important characteristics that could affect outcomes (e.g., demographics, risk factors, co-morbid conditions)? 7. Was the overall drop-out rate from the study at endpoint 20% or lower than the number allocated to treatment? 8. Was the differential drop-out rate (between treatment groups) at endpoint 15 percentage points or lower? 9. Was there high adherence to the intervention protocols for each treatment group? 10. Were other interventions avoided or similar in the groups (e.g., similar background treatments)? 11. Were outcomes assessed using valid and reliable measures, implemented consistently across all study participants? 12. Did the authors report that the sample size was sufficiently large to be able to detect a difference in the main outcome between groups with at least 80% power? 13. Were outcomes reported or subgroups analyzed prespecified (i.e., identified before analyses were conducted)? 14. Were all randomized participants analyzed in the group to which they were originally assigned; i.e., did they use an intention-to-treat analysis? N: No. NA: Not Applicable. NR: Not Reported. Y: Yes. Item scores of N and NR increased risk of bias. Overall Quality (Risk of Bias) Rating: [22] 0–4: Poor: high risk of bias (**High**). 5–9: Fair—between low and high risk of bias, some concerns (**Some**). 10–14: Good—low risk of bias (**Low**).

**Table 4:** Empirical evidence statements for functional biomarkers and risk of low back pain.

Functional Biomarker	Empirical Evidence Statement	Study Findings	Conclusion	Confidence Level
Relational:				
Aerobic Capacity	Poor aerobic capacity is associated with increased risk of LBP	Mixed: 2 studies of fair quality [46,47].	Inconclusive	Low
Anthropometric	Obesity is associated with increased risk of LBP	Support: 3 studies—2 of fair quality [6,7]; 1 of poor quality [36]. Against: 5 studies—3 of fair quality [35,43,46]; 2 of poor quality [38,39].	Inconclusive	Moderate
Disability/Kinesiophobia	Higher level of disability/kinesiophobia is associated with increased risk of LBP	Support: 3 studies of fair quality [7,40,48]. Mixed: 1 study of fair quality [6].	Support	Moderate
Functional Work Tasks/Capacity	Poor performance on functional work tasks/capacity is associated with increased risk of LBP	Support: 5 studies—3 of fair quality [7,40,44]; 2 of poor quality [36,39]. Mixed: 1 study of fair quality [47].	Support	Moderate
Imaging/Structural/Morphological	Abnormal imaging/structural/morphological findings in the lumbar spine are associated with increased risk of LBP	Mixed: 1 study of fair quality [41].	Inconclusive	Insufficient Evidence
Kinematics	Abnormal trunk kinematics are associated with increased risk of LBP	Mixed: 1 study of fair quality [45]. Against: 1 study of fair quality [42].	Inconclusive	Low
Movement Quality/Range of Motion	Poor movement quality/range of motion is associated with increased risk of LBP	Support: 1 study of fair quality [45]. Against: 2 studies of fair quality [7,46].	Against	Low
Muscular Fitness	Poor muscular fitness is associated with increased risk of LBP	Mixed: 3 studies of fair quality [6,7,46]. Against: 1 study of fair quality [42].	Inconclusive	Moderate
Overall Physical Fitness	Poor overall physical fitness is associated with increased risk of LBP	Support: 1 study of fair quality [34]. Against: 1 study of fair quality [35].	Inconclusive	Low
Physical Activity	Low level of physical activity is associated with increased risk of LBP	Support: 2 studies—1 of fair quality [7]; 1 of poor quality [39]. Mixed: 1 study of fair quality [46]. Against: 3 studies—1 of fair quality [40]; 2 of poor quality [36,37].	Inconclusive	Low
Interventional:				
Muscular Fitness	Exercise interventions targeting trunk muscular fitness, alone or combined with movement quality/range of motion, are beneficial for improving LBP clinical outcomes	Support: 2 studies of fair–good quality [6,12].	Support	Low

Key: LBP: low back pain. Study findings—Support: All comparisons in the study support the empirical evidence statement (ESS). Mixed: Some comparisons in the study support the ESS, while other comparisons do not support the ESS. Against: all comparisons in the study do not support ESS. Conclusion—Support: ≥Two-thirds of fair–good quality studies support the ESS. Against: ≥Two–thirds of fair–good quality studies do not support the ESS. Inconclusive: Studies do not meet criteria for Support or Against categories. Confidence level—Insufficient Evidence: This includes evidence from ≤1 study of fair–good quality. Low: This includes evidence from 2–3 studies of fair–good quality. Moderate: This includes evidence from 4–5 studies of fair–good quality. High: This includes evidence from ≥6 studies of fair–good quality.

## Data Availability

The original contributions presented in this study are included in the article. Further inquiries can be directed to the corresponding author.

## Appendix A

Table A1: PRISMA checklist; Table A2: PubMed search strategy.

**Table A1:** PRISMA checklist.

Section and Topic	Item Number	Checklist Item	Location Where Item Is Reported
**TITLE: Functional Biomarkers Associated with Risk of Low Back Pain in Firefighters: A Systematic Review**			**Page Number**
Title	1	Identify the report as a systematic review.	1
**ABSTRACT**			
Abstract	2	See the PRISMA 2020 for Abstracts checklist.	1
**INTRODUCTION**			
Rationale	3	Describe the rationale for the review in the context of existing knowledge.	1–2
Objectives	4	Provide an explicit statement of the objective(s) or question(s) the review addresses.	2
**METHODS**			
Eligibility criteria	5	Specify the inclusion and exclusion criteria for the review and how studies were grouped for the syntheses.	3–4
Information sources	6	Specify all databases, registers, websites, organisations, reference lists and other sources searched or consulted to identify studies. Specify the date when each source was last searched or consulted.	4
Search strategy	7	Present the full search strategies for all databases, registers and websites, including any filters and limits used.	Table A2—Appendix A
Selection process	8	Specify the methods used to decide whether a study met the inclusion criteria of the review, including how many reviewers screened each record and each report retrieved, whether they worked independently, and if applicable, details of automation tools used in the process.	4–5
Data collection process	9	Specify the methods used to collect data from reports, including how many reviewers collected data from each report, whether they worked independently, any processes for obtaining or confirming data from study investigators, and if applicable, details of automation tools used in the process.	5
Data items	10a	List and define all outcomes for which data were sought. Specify whether all results that were compatible with each outcome domain in each study were sought (e.g., for all measures, time points, analyses), and if not, the methods used to decide which results to collect.	3–4, Table 1 and Table 2
	10b	List and define all other variables for which data were sought (e.g., participant and intervention characteristics, funding sources). Describe any assumptions made about any missing or unclear information.	3–4, Table 1 and Table 2
Study risk of bias assessment	11	Specify the methods used to assess risk of bias in the included studies, including details of the tool(s) used, how many reviewers assessed each study and whether they worked independently, and if applicable, details of automation tools used in the process.	5, Table 3
Effect measures	12	Specify for each outcome the effect measure(s) (e.g., risk ratio, mean difference) used in the synthesis or presentation of results.	5–6
Synthesis methods	13a	Describe the processes used to decide which studies were eligible for each synthesis (e.g., tabulating the study intervention characteristics and comparing against the planned groups for each synthesis (item #5)).	7
	13b	Describe any methods required to prepare the data for presentation or synthesis, such as handling of missing summary statistics, or data conversions.	4–5
	13c	Describe any methods used to tabulate or visually display results of individual studies and syntheses.	4–5, Table 1, Table 2 and Table 4
	13d	Describe any methods used to synthesize results and provide a rationale for the choice(s). If meta-analysis was performed, describe the model(s), method(s) to identify the presence and extent of statistical heterogeneity, and software package(s) used.	4–5, 7
	13e	Describe any methods used to explore possible causes of heterogeneity among study results (e.g., subgroup analysis, meta-regression).	5–6
	13f	Describe any sensitivity analyses conducted to assess robustness of the synthesized results.	5–6
Reporting bias assessment	14	Describe any methods used to assess risk of bias due to missing results in a synthesis (arising from reporting biases).	5
Certainty assessment	15	Describe any methods used to assess certainty (or confidence) in the body of evidence for an outcome.	7, Table 4
**RESULTS**			
Study selection	16a	Describe the results of the search and selection process, from the number of records identified in the search to the number of studies included in the review, ideally using a flow diagram.	7, Figure 1
	16b	Cite studies that might appear to meet the inclusion criteria, but which were excluded, and explain why they were excluded.	7, Figure 1
Study characteristics	17	Cite each included study and present its characteristics.	8–9, Table 1 and Table 2
Risk of bias in studies	18	Present assessments of risk of bias for each included study.	17, Table 3
Results of individual studies	19	For all outcomes, present, for each study: (a) summary statistics for each group (where appropriate) and (b) an effect estimate and its precision (e.g., confidence/credible interval), ideally using structured tables or plots.	Table 2
Results of syntheses	20a	For each synthesis, briefly summarise the characteristics and risk of bias among contributing studies.	20, Table 4
	20b	Present results of all statistical syntheses conducted. If meta-analysis was done, present for each the summary estimate and its precision (e.g., confidence/credible interval) and measures of statistical heterogeneity. If comparing groups, describe the direction of the effect.	Table 2 and Table 4
	20c	Present results of all investigations of possible causes of heterogeneity among study results.	not applicable
	20d	Present results of all sensitivity analyses conducted to assess the robustness of the synthesized results.	not applicable
Reporting biases	21	Present assessments of risk of bias due to missing results (arising from reporting biases) for each synthesis assessed.	not applicable
Certainty of evidence	22	Present assessments of certainty (or confidence) in the body of evidence for each outcome assessed.	20, Table 4
**DISCUSSION**			
Discussion	23a	Provide a general interpretation of the results in the context of other evidence.	23
	23b	Discuss any limitations of the evidence included in the review.	23–24
	23c	Discuss any limitations of the review processes used.	not applicable
	23d	Discuss implications of the results for practice, policy, and future research.	24–25
**OTHER INFORMATION**			
Registration and protocol	24a	Provide registration information for the review, including register name and registration number, or state that the review was not registered.	3
	24b	Indicate where the review protocol can be accessed, or state that a protocol was not prepared.	3
	24c	Describe and explain any amendments to information provided at registration or in the protocol.	3
Support	25	Describe sources of financial or non-financial support for the review, and the role of the funders or sponsors in the review.	25
Competing interests	26	Declare any competing interests of review authors.	26
Availability of data, code and other materials	27	Report which of the following are publicly available and where they can be found: template data collection forms; data extracted from included studies; data used for all analyses; analytic code; any other materials used in the review.	25

**Table A2:** PubMed search strategy.

((“Firefighters”[Mesh] OR “firefighter*”) OR (“Fires”[Mesh] or “fire*”)) AND ((“Occupational Injuries”[Mesh] OR “occupational injur*”) OR (“Occupational Diseases”[Mesh] OR “occupational disease*”) OR (“Wounds and Injuries”[Mesh] OR “wound*” OR “injur*”) OR (“Biomechanical Phenomena”[Mesh] OR “biomechanic*” OR “kinematic*”) OR (“Electromyography”[Mesh] OR “Electromyography”) OR (“Diagnostic Imaging”[Mesh] OR “diagnostic imag*”) OR (“Muscles”[Mesh] OR “muscle”) OR (“Exercise Test”[Mesh] OR “exercise test”) OR (“Physical Fitness”[Mesh] OR “physical fitness”) OR (“Physical Functional Performance”[Mesh] OR “physical functional performance” OR “functional performance” OR “physical performance”) OR (“Persons with Disabilities”[Mesh] OR “disabilit*” OR “disabled” OR “disabil*”) OR (“Body Composition”[Mesh] OR “body composition”) OR (“Body Mass Index”[Mesh] OR “body mass index” OR “BMI”) OR (“Waist Circumference”[Mesh] OR “waist circumference”)) AND ((“Back Injuries”[Mesh] OR “back injur*” OR “back”) OR (“Thoracic Injuries”[Mesh] OR “thoracic injur*”) OR (“Thoracic Vertebrae”[Mesh] OR “Thoracic Vertebrae”) OR (“Thorax”[Mesh] OR “thorax”) OR (“Sacrum”[Mesh] OR “sacrum”) OR (“Back Pain”[Mesh] OR “back pain” OR “dorsalgia”) OR (“Low Back Pain”[Mesh] OR “low back pain” OR “lumbago”) OR (“Coccyx”[Mesh] OR “coccyx”) OR (“Sciatica”[Mesh] OR “sciatic*” OR “sciatic neuropathy”) OR (“Spondylosis”[Mesh] OR “spondylosis”) OR (“back disorder*”) OR (“Spine”[Mesh] OR “spine”) OR (“Discitis”[Mesh] OR “discitis”) OR (“Spinal Diseases”[Mesh] OR “spinal disease*”) OR (“Intervertebral Disc Degeneration”[Mesh] OR “Intervertebral Disc Degeneration” OR “disc prolapse”) OR (“Intervertebral Disc Displacement”[Mesh] OR “Intervertebral Disc Displacement” OR “disc herniation”) OR (“Spinal Fusion”[Mesh] OR “spinal fusion”) OR (“Spinal Neoplasms”[Mesh] OR “spinal neoplasm*”) OR (“Zygapophyseal Joint”[Mesh] OR “Zygapophyseal Joint”) OR (“Intervertebral Disc”[Mesh] OR “Intervertebral Disc”) OR (“Intervertebral Disc Displacement”[Mesh] OR “Intervertebral Disc Displacement”) OR (“Post-laminectomy syndrome” OR “postlaminectomy”) OR (“Arachnoiditis”[Mesh] OR “Arachnoiditis”) OR (“Failed Back Surgery Syndrome”[Mesh] OR “failed back surgery syndrome” OR “failed back surger*” OR “failed back”))
